# Real‐World Data on Inotuzumab Ozogamicin for Adult Patients With Relapsed/Refractory Acute Lymphoblastic Leukemia: A GRELAL‐Chile Study

**DOI:** 10.1002/cam4.71230

**Published:** 2025-09-12

**Authors:** Marcela Espinoza, Jorge Rojas‐Vallejos, Nicolás Rodríguez, Gonzalo Guerrero, Miguel López, Natalia Aranguiz, Guillermo Conte, Francisco Samaniego, Nicolás Quinteros, Daniel Astete, Lucas Carcamo, Constanza Flores, Ximena Huerta, Mauricio Chandía, Jorge Valenzuela, Marcelo Navarrete, Yorman Flores, Agatha Larrazabal, Edgar Zapata, Joaquín Jerez

**Affiliations:** ^1^ Clínica Dávila Santiago Chile; ^2^ Universidad Andres Bello Santiago Chile; ^3^ Hospital Barros Luco Trudeau Santiago Chile; ^4^ Clínica RedSalud Vitacura Santiago Chile; ^5^ Clínica Alemana Santiago Chile; ^6^ Hospital Clínico de la Universidad de Chile Santiago Chile; ^7^ Departamento Medicina Interna Sede Centro Universidad de Chile Hospital Clínico San Borja de Arriarán Santiago Chile; ^8^ Hospital Gustavo Fricke Viña del Mar Chile; ^9^ Hospital San Juan de Dios de La Serena La Serena Chile; ^10^ Hospital Carlos Van Buren Valparaíso Chile; ^11^ Hospital Guillermo Gran Benavente Concepción Chile; ^12^ Universidad de Concepción Concepción Chile; ^13^ Universidad de Magallanes Punta Arenas Chile; ^14^ Pontificia Universidad Católica de Chile Santiago Chile; ^15^ Hematology Resident Universidad de los Andes Santiago Chile; ^16^ Programa de Doctorado en Biomedicina, Facultad de Medicina Universidad de los Andes Santiago Chile

**Keywords:** acute lymphoblastic leukemia, Inotuzumab Ozogamicin, sinusoidal obstruction syndrome

## Abstract

**Background:**

Inotuzumab Ozogamicin (InO) has shown efficacy in relapsed/refractory acute lymphoblastic leukemia (R/R ALL), but evidence from Latin America is scarce. We evaluated the outcomes of Chilean patients treated with InO in public and private health centers.

**Methods:**

We retrospectively analyzed 35 patients with R/R ALL (median age 33 years; 54% male). Twenty percent had BCR::ABL‐positive ALL, 78% expressed CD22, and 91% expressed CD19. Response rates, measurable residual disease (MRD), survival outcomes, and treatment‐related toxicities were assessed. Multivariate analyses explored prognostic factors.

**Results:**

Complete remission or remission with incomplete recovery (CR/CRi) was achieved in 74% of patients. Among those evaluated, 82% reached MRD < 0.01%. Patients undergoing allogeneic hematopoietic stem cell transplantation (Allo‐HSCT) after InO had superior overall survival (OS) compared with those who did not (24.2 vs. 5.2 months). Median progression‐free survival (PFS) was 6.9 months and median OS was 8.8 months. Sinusoidal obstruction syndrome occurred in 14% of patients but was generally mild. Multivariate analysis identified comorbidities and high blast counts as adverse prognostic factors, whereas MRD negativity and subsequent Allo‐HSCT were associated with improved outcomes.

**Conclusions:**

InO demonstrated high remission and MRD negativity rates in Chilean patients with R/R ALL, with OS and PFS comparable to existing research. Although SOS incidence was higher, it was generally mild. Achieving MRD negativity and proceeding to Allo‐HSCT provided the greatest survival benefit. Study limitations include short follow‐up and limited data.

## Graphical abstract

This study assesses the outcomes of Inotuzumab Ozogamicin (InO) in 35 Chilean patients with relapsed/refractory acute lymphoblastic leukemia (R/R ALL) treated in public and private health centers. A complete response was achieved in 74% of patients, with improved overall survival in those undergoing allogeneic stem cell transplants. While survival outcomes were comparable to previous studies, a higher incidence of veno‐occlusive disease was observed.

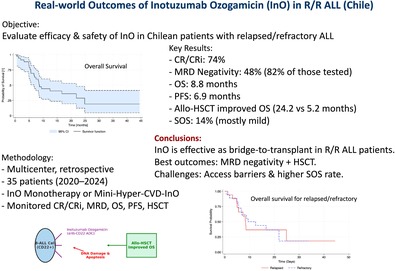

## 1 Introduction

Acute Lymphoblastic Leukemia (ALL) accounts for 20% of adult leukemia cases, with over 90% achieving complete remission (CR) after first‐line polychemotherapy [1, 2, 3]. However, 10%–20% show primary resistance, and 30%–60% relapse based on risk factors [4]. Prognosis for relapsed/refractory ALL (R/R ALL) remains poor, with a median overall survival (OS) of 4.5 to 6 months, with survival rates at 5 years lower than 10% [1, 5]. Historically, rescue polychemotherapy with conventional cytotoxic agents, combined with Allogeneic Hematopoietic Stem Cell Transplantation (Allo‐HSCT), was the standard treatment for R/R ALL, but long‐term remission is achieved in only 30%–40% of patients, depending on risk factors [6, 7, 8].

Inotuzumab Ozogamicin (InO), a monoclonal IgG antibody that targets CD22 on malignant B cells inducing double‐strand DNA breaks [9], was approved by the European Medicine Agency (EMA) for use in adult patients with relapsed/refractory ALL in June 2017 [10], incorporated into the National Institute for Health and Care Excellence (NICE) guidelines in the UK in 2019 [11], and approved by the Chilean Public Health Institute in July 2019 [12]. InO provides significant CR rates with a manageable toxicity profile in patients with R/R ALL. Nonetheless, its use has challenges, most notably because of the risk of developing hepatotoxicity, including Sinusoidal Obstruction Syndrome (SOS) when undergoing Allo‐HSCT [13]. In resource‐limited countries, access to InO remains a significant barrier [14, 15]. These challenges highlight the need for careful patient selection, rigorous monitoring, and efforts to improve equitable access in both public and private healthcare systems.

Although InO has been available in the Chilean market since 2019, it is not yet included in the country's treatment guidelines. This study aims to evaluate the use, efficacy, and safety of InO in patients with R/R ALL enrolled in the National ALL Registry (GRELAL, for its acronym in Spanish) in Chile, a population underrepresented in the scientific literature.

### 1.1 Study Design

This is an observational, retrospective, multicenter study. It includes patients over 15 years old with R/R ALL, either BCR::ABL1 positive or negative, who were indicated for the use of InO. Medical records from January 1, 2020, to July 31, 2024, were reviewed. All centers had the ethics committee's approval for the study. A total of 35 cases from 8 institutions were collected (27 patients from private clinics and eight from public hospitals). Written consent was obtained from living patients at the time of data collection, while the Ethics Committee granted a consent exemption for deceased patients.

### 1.2 Patients

Adult patients aged 15 years or older with R/R ALL were included. Flow cytometry confirmed the diagnosis, which showed the presence of B‐lymphoblasts. Patients with a lymphoid blast phase of Chronic Myeloid Leukemia were also included. Patients with significant liver disease (bilirubin > 2) or a history of SOS were excluded. Tables 1 and 2 report the most relevant characteristics of these patients.

**TABLE 1:** Descriptive statistics for laboratory variables for all individuals.

Variable name	Obs	Mean	SD	Min	Max
Age at diagnosis (years)	35	33.2	16.3	9.7	73.6
Hb (g/dL)	32	8.8	2.5	3.3	14.7
WBC (cells/mL)	33	38,984	73,933	500	285,900
Blast PB (cells/mL)	31	24,497	54,579	0	235,800
PLT (cells/mL)	32	95,844	95,338	7000	430,000
Blast BM (%)	25	74	36	2	100
AST U/L	30	47	29	9	142
ALT U/L	25	72	78	9	292
Bilirubin (mg/dL)	30	0.54	0.38	0.13	1.49
Creatinine (mg/dL)	28	0.87	0.27	0.48	1.50

Abbreviations: ALT, alanine aminotransferase; AST, aspartate aminotransferase; BM, bone marrow; Hb, hemoglobin; PB, peripheral blood; PLT, platelets; WBC, white blood cells.

**TABLE 2:** Sample composition.

	Value
Characteristics	
Male	19/35 (54.3%)
Private centers	28/35 (80%)
BCR::ABL1 positive	7/35 (20%)
Immunophenotype	
Common	30/35 (85%)
Pre‐B	2/35 (5.7%)
Pro‐B	1/35 (2.9%)
Burkitt	1/35 (2.9%)
Mixed phenotype acute leukemia	1/35 (2.9%)
Karyotype at diagnosis	
*t*(9;22)	7/35 (20%)
*t*(4;11)	1/35 (2.9%)
*t*(1;19)	1/35 (2.9%)
*t*(8;22)	1/35 (2.9%)
Complex	3/35 (8.6%)
Normal	17/35 (45.7%)
Unknown	5/35 (14.3%)
N° of prior therapeutic lines	
1	10/35 (28.6%)
2	5/35 (14.3%)
> 2	20/35 (57.1%)
Allo‐HSCT prior to InO	5/35 (14.3%)
Duration of 1st CR prior to InO	
< 12 m	13/17 (76.5%)
> 12 m	4/17 (23.5%)
Response to pre‐InO lines	
Relapsed	17/35 (48.6%)
Resistant/Refractory	18/35 (51.4%)
Median follow‐up (months) after InO (min, max)	9.5 months (0.2–42)

### 1.3 Treatment

Patients received intravenous InO following one of two protocols based on the treating physician's choice: (i) InO Monotherapy, as in INO‐VATE protocol [14]: Initial dose of InO was 1.8 mg/m^2^ per cycle every 28 days, administered as 0.8 mg/m^2^ on day 1 and 0.5 mg/m^2^ on days 8 and 15. Subsequent cycles consisted of 0.5 mg/m^2^ on days 1, 8, and 15 every 28 days, for up to 3 cycles. Response to therapy was monitored by bone marrow aspirate (BMA) analysis and MRD. Response assessment was done at the end of cycle 1 and after cycle 2. (ii) Mini‐Hyper‐CVD‐InO [16]: Odd cycles: InO at 1.3 mg/m^2^ on day 3. Dexamethasone 20 mg on days 1–4 and 11–12. Cyclophosphamide 150 mg/m^2^ on days 1, 2, and 3. Vincristine 2 mg on days 1 and 8. Even cycles: InO at 1.3 mg/m^2^ accompanied by Methotrexate 250 mg/m^2^ on day 1 and Cytarabine 500 mg/m^2^ every 12 h on days 2 and 3, for up to 8 cycles. All patients received intrathecal chemotherapy with Cytarabine, Methotrexate, and Betamethasone. Response assessment was done at the end of cycle one and after 2–4 cycles.

### 1.4 Outcomes

The primary objective was to evaluate the efficacy and safety of InO in patients with R/R ALL. Key endpoints included complete response (CR) with or without hematologic recovery, duration of response (DOR), progression‐free survival (PFS), overall survival (OS), and the completion of allogeneic hematopoietic stem cell transplantation (Allo‐HSCT) following InO use.

Complete response (CR) is defined as fewer than 5% blasts in bone marrow, blood neutrophil count > 1000 cells/mL, platelet count > 100 cells/mL, and absence of extramedullary involvement. Incomplete CR (CRi) is defined as meeting CR criteria but with an absolute neutrophil count (ANC) < 1000 cells/mL, platelet count < 100 cells/mL, or both. Partial response: decrease in bone marrow blasts by 50% but remaining in the 6%–25% range. Early death is defined as death occurring before meeting CR criteria or in cases of resistant disease. Duration of CR corresponds to the time from the first CR to relapse or the last follow‐up in CR. Measurable residual disease (MRD) negativity is defined as bone marrow blasts < 0.01%, measured by multiparametric flow cytometry. Overall survival (OS) corresponds to the time from the first InO infusion to death or the last follow‐up. Progression‐free survival (PFS) corresponds to the time from the first InO infusion to evidence of progression.

### 1.5 Adverse Events

Adverse events were defined as any event occurring between the first dose and 6 weeks after the last dose of InO. All cases of SOS occurring within the first 2 years were considered adverse events. Toxicity grading followed the Common Terminology Criteria for Adverse Events (CTCAE) v4.03.

### 1.6 Statistical Analysis

Descriptive statistics summarize key patient characteristics, including mean values, standard deviations, frequency, and extreme values for clinical and laboratory variables. Kaplan–Meier survival analysis estimates overall survival (OS) and progression‐free survival (PFS), with log‐rank tests used to compare survival outcomes between different patient subgroups. Additionally, for the multivariate Cox regression models, covariates were selected based on a combination of statistical significance in univariate analysis, clinical relevance, and theoretical considerations. Variables such as comorbidity, percentage of blasts, and BCR::ABL1 status were included not only due to their statistical associations, but also because of their established prognostic value in relapsed/refractory ALL. Age and sex were retained to account for potential confounding. To assess the stability of our findings and avoid overfitting, especially given the small sample size (*n* = 35), we estimated multiple Cox models with different covariate combinations. This approach allowed us to explore how the inclusion or exclusion of variables with missing data or borderline significance affected the magnitude and direction of key hazard ratios. Last, we constructed a blood index using Principal Component Analysis (PCA) to capture a composite measure of hematologic status at baseline. Five laboratory variables were included: hemoglobin, hematocrit, platelet count, white blood cell count, and neutrophil count. These variables were first standardized into *z*‐scores, and PCA was then applied to the correlation matrix. The first principal component (PC1), which explained the largest proportion of shared variance, was extracted and used as the blood index in the regression analyzes.

Because both MRD negativity and Allo‐HSCT occur after treatment initiation, they are inherently time‐dependent events. However, the exact date of MRD negativity was unavailable in our dataset, so MRD was analyzed as a fixed post‐treatment variable. We acknowledge that this may introduce immortal time bias and interpret the results accordingly. In the case of Allo‐HSCT, transplant dates were available, but survival comparisons were conducted descriptively rather than within time‐dependent models. We intended to report clinical outcomes as observed, rather than estimate causal effects.

## 2 Results

From January 2020 to July 2024, 35 patients from public and private centers were included in the study, and their characteristics are shown in Tables 1 and 2. Seven patients had BCR::ABL1‐positive ALL, and 17 had a normal karyotype. 85% were common B‐ALL, CD22 status was reported in 19 patients and was positive in 78%. CD19 was assessed in all patients and was positive in 91.4%. This analysis included one patient with mixed‐lineage leukemia (MPAL), one patient with B‐lymphoblastic lymphoma (B‐LBL), and one patient with Lymphoid Blast Crisis of Chronic Myeloid Leukemia. All of them were CD22 positive. Seventeen patients had relapsed disease. Regarding the duration of the first CR, it was less than 12 months in 13 patients. Before rescue treatment with InO, 20 had received three or more prior therapies, and five had received Allo‐HSCT.

### 2.1 Treatment

Regarding treatment with InO, 31 patients received InO monotherapy, and 4 received Mini‐Hyper‐CVD‐InO. Among the 7 BCR::ABL1‐positive patients, the combined Tyrosine Kinase Inhibitors were Dasatinib in 5 cases and Ponatinib in 2. The maximum cycles received in InO Monotherapy were a maximum of 3 (6 patients one cycle, 18 patients two cycles, and two patients three cycles). Regarding Mini‐Hyper‐CVAD‐InO, five patients reached Phase IA, and responses were assessed. Three patients were in Phase IB, and one was in Phase IIB. After a median of 2 cycles of InO (range 1–3 cycles), 26 patients (74%) achieved CR/CRi (20 CR and 6 CRi), and 5 showed partial response or remained refractory. MRD was measured in 29 patients, 14 of whom (48%) achieved MRD negativity. The median follow‐up for the series was 9.3 months (range 0.2–42 months). Four patients experienced early death without knowing the status of their disease (3 due to infection/septic shock, including one case of COVID‐19 pneumonia and one suicide).

Five patients received InO for post‐transplant relapse. Three were refractory, while two achieved CR; one of them proceeded to transplantation but died of severe SOS, and another achieved CR but was not eligible by the Health Care insurance for a second transplant and died from disease progression. Regarding patients who also received blinatumomab, a total of four cases were reported: two received blinatumomab before InO without achieving CR but achieved it with InO, one received it after InO and achieved MRD negativity, enabling transplantation, and another received it as a rescue post‐InO and post‐transplant but did not achieve CR.

Regarding transplantation, conditioning was myeloablative in 16 patients (80%) and of reduced intensity (RI) in 4 (20%). The most commonly used conditioning regimen was fludarabine (Flu) + total body irradiation (TBI) in 16 cases, Flu + TBI‐200 + cyclophosphamide in 3 cases, and melphalan + Flu + TBI‐200 in 1 case. Most transplants (16/20) were haploidentical donor transplants; four were fully matched related donor transplants. One patient received a second transplant. None of the patients received CAR‐T therapy. Among the transplanted patients, three died before the first evaluation: 2 from septic shock and one from COVID‐19 pneumonia. Six out of 20 patients experienced disease progression after the first post‐transplant evaluation.

### 2.2 Survival

**FIGURE 1:**
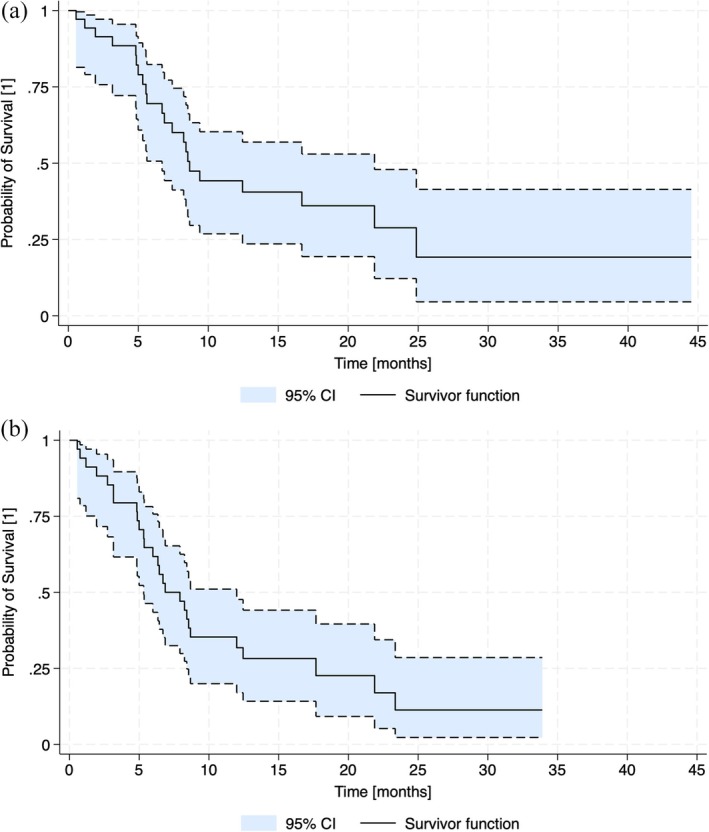
Survival curves for all individuals in the sample. (a) Overall survival. (b) Progression‐free survival.

The duration of response was 10.9 months (95% CI 19.6–22.‐8). Figure 1a shows the overall survival, which was 8.8 months (95% CI 15.9–18.3), and progression‐free survival (Figure 1b), which was 6.9 months (95% CI 15.2–17.5). Among the four patients who received blinatumomab at any point, their overall survival was not significantly different from the rest (*p* = 0.5).

**FIGURE 2:**
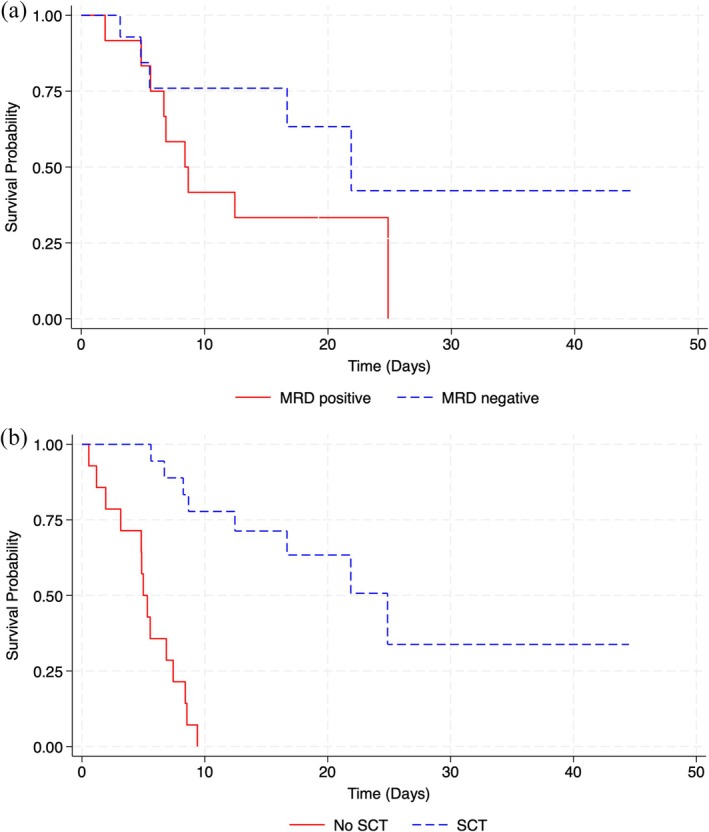
Survival curves for all individuals with CR/CRi. (a) Overall survival for MRD. (b) Overall survival for Allo‐HSCT.

The median overall survival of the 26 patients in CR/CRi differed between those who achieved MRD negativity (22.2 months) and those with MRD positivity (10.7 months). However, the difference was not statistically significant (95% CI 0.6–6.7, *p* = 0.1) (Figure 2a). A slightly better response was observed in MRD‐negative refractory patients compared to MRD‐negative relapsed ones, but this difference was also not statistically significant.

Patients who underwent Allo‐HSCT (Figure 2b) had a higher overall survival (OS) of 25.7 months compared to those who did not undergo Allo‐HSCT, with an OS of 5.2 months (95% CI 1.9–11.89 months, *p* < 0.001). The OS of patients who received Allo‐HSCT after InO was 24.2 months.

**FIGURE 3:**
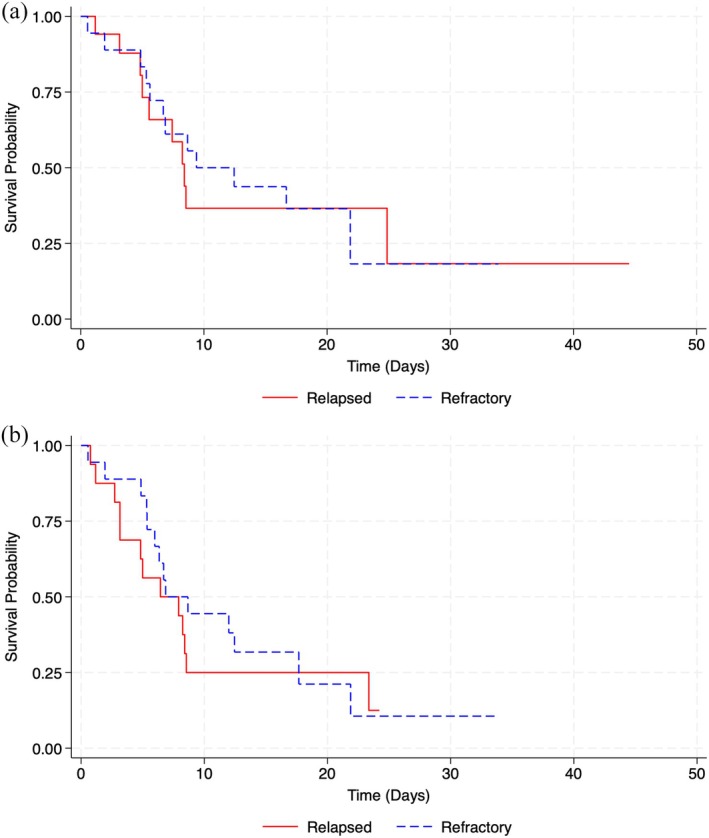
Survival curves according to refractory/relapsed patients. (a) Overall survival for relapsed/refractory. (b) Progression‐free survival for relapsed/refractory.

Regarding relapsed and refractory patients, progression‐free survival (PFS) was 6.5 and 7.9 months (95% CI 0.8–2.3), and overall survival (OS) was 8.5 and 9.5 months (95% CI 0.38–3.09). The two groups had no significant differences (Figure 3a,b).

### 2.3 Safety and Adverse Events

Table 3 shows the adverse events reported and their grade. During treatment with InO, 18 adverse events were recorded and 20 in patients who underwent Allo‐HSCT. During InO treatment, the most common adverse event was infection, mainly pneumonia, one related to COVID‐19. Regarding transplant, the most common adverse event was febrile neutropenia in 10 patients. Hematological adverse events were common in all patients, so they were not reported.

**TABLE 3:** Adverse events reported.

Adverse event grade	1–2	3–4	5
InO treatment			
Infection	8	2	0
Sepsis	—	0	1
Hemorrhage	1	0	0
SOS	2	0	0
DILI	4	0	0
Transplant			
GVHD			
Intestinal	2	1	0
Hepatic	1	0	0
Bronchial infection	0	0	1
CMV reactivation	1	0	0
BK virus reactivation	0	1	0
Febrile neutropenia	—	10	0
Sepsis	—	0	1
DILI	1	0	0
SOS	4	0	1

*Note:* Common adverse events reported during InO treatment and stem cell transplantation.

Abbreviations: CMV, cytomegalovirus; DILI, drug‐induced liver injury; GVHD, graft versus host disease; SOS, sinusoidal obstruction syndrome.

Regarding SOS, there were 7 cases (7/35): 2 during InO administration (both grade 2) and five during Allo‐HSCT (5/20) (four grade 2 and one grade 5). Six of these seven patients received two cycles of InO, and one patient received three. The fatal SOS case was a patient with Burkitt LBL who received two cycles of InO before the first Allo‐SCT and one cycle before the second Allo‐SCT. None of the patients had received dual alkylating agents, all were under 50 years of age, and none received defibrotide. The patients responded to medical management and were treated with ursodeoxycholic acid. Patients with SOS did not have significant liver function abnormalities before InO administration.

### 2.4 Multivariate Analysis

Table 4 presents five Cox proportional hazard models, each estimated using a different set of covariates. These models were specified to assess the robustness of the associations between key predictors and overall survival under varying conditions. By including or excluding variables based on availability and relevance, we aimed to evaluate the consistency of hazard ratios across models.

**TABLE 4:** Cox hazard models with varying covariate specifications for overall survival.

	(1) Full model	(2)	(3)	(4)	(5) Preferred
Female	1.610	0.175			
	(1.096)	(0.218)			
Age at diagnosis	0.990	1.014	0.997		
	(0.019)	(0.031)	(0.018)		
Blood index	1.529	7.634***	1.390	1.385	
	(0.415)	(5.836)	(0.344)	(0.341)	
% of blasts	1.027***	1.113**	1.026**	1.026***	1.025***
	(0.011)	(0.043)	(0.010)	(0.010)	(0.009)
SOS InO	0.387				
	(0.346)				
SOS Allo HSCT		97.558**			
		(221.187)			
BCR::ABL1	0.343	0.255	0.358	0.362	0.362*
	(0.301)	(0.310)	(0.269)	(0.271)	(0.225)
Comorbidity	6.863***	2.553	5.710***	5.497***	4.437**
	(5.182)	(2.843)	(3.837)	(3.472)	(2.589)
Observations	24	16	24	24	29

*Note:* Standard errors are reported in parentheses. All specifications are Cox regressions with no ties. Hazard ratios are reported. Each column represents an independent model estimated to assess the robustness of key predictors under alternative variable inclusion criteria. Model (5) is used for primary interpretation. The blood index is built using principal component analysis (PCA) as described in the Method section. Female, SOS InO, SOS Allo‐HSCT, BCR::ABL1, and Comorbidity are dummy variables; age at diagnosis is measured in years, and % of blasts is in percentage.

* *p* < 0.10.

** *p* < 0.05.

*** *p* < 0.01.

Table 4 presents results from Cox proportional hazards regressions showing how various covariates influence the hazard rate, in this case, the risk of death. The hazard ratios indicate the multiplicative effect of each covariate on the hazard. Statistically significant variables include the percentage of blasts, which consistently increases the hazard by around 2.5% across all models, and comorbidity, which also shows a strong and significant association with higher hazard rates, increasing the risk of death by about 4 times. The blood index, created using principal component analysis (PCA) of blood‐related variables, is statistically significant in one model but is not consistently significant across others, suggesting high sensitivity to model specification.

Non‐significant covariates include the dummy variable female and age at diagnosis, which consistently did not show meaningful effects on the hazard. Whether the patient was BCR:: ABL1 positive or not, it exhibited a potential protective effect by reducing the risk of death by about 60%. Still, it is only weakly significant in the column (5) model specification. The results highlight differences across models, possibly due to variations in sample size and covariate inclusion. Overall, the analysis underscores the importance of the percentage of blasts and comorbidity as predictors of hazard while emphasizing the need for careful interpretation of results due to variability in significance across models. Nonetheless, future research with a larger sample size may be fruitful in studying models considering the variables used in Table 4.

## 3 Discussion

We present real‐world experience with InO in patients with R/R ALL in Chile, where access barriers, patient heterogeneity, and limited public insurance coverage influence treatment decisions. The median age of our cohort was younger than in previous reports (32 vs. 50 years) [17, 18], likely reflecting prioritization of adolescent and young adult (AYA) patients in a resource‐constrained context. Notably, 80% of patients were treated in private centers, limiting generalizability to Chile's broader population, of whom 83% rely on the public health system. Our cohort also included patients with MPAL and B‐LBL subtypes, who are typically excluded from trials; their responses varied, consistent with their biological heterogeneity.

The observed CR/CRi rate of 74% aligns with prior studies [17, 18]. Over half of our patients had received three or more prior therapies, and 14% had undergone Allo‐HSCT before InO, underscoring the high‐risk nature of this group. While some patients received InO post‐HSCT in a palliative setting, this likely reflects the unavailability of CAR‐T therapy [19, 20] in Chile. Patients who underwent Allo‐HSCT after InO had markedly better overall survival (24.2 months), echoing the pattern reported by Marks et al. [13]. Survival appeared even better in those transplanted with MRD negativity, although MRD was modeled as a fixed post‐treatment variable due to missing date information. As discussed in the Methods, this introduces potential immortal time bias and limits causal interpretation. Although based on actual timing, Allo‐HSCT comparisons were also presented descriptively rather than through time‐dependent modeling.

In line with previous evidence, MRD negativity and blinatumomab exposure were associated with favorable responses [21, 22, 23], though optimal sequencing and regimen combinations remain under investigation [24]. InO appears to be particularly effective as a bridge to transplant in resource‐limited environments. A Mini‐CVAD + InO + blinatumomab strategy may serve as a viable alternative when Allo‐HSCT is not feasible, with survival outcomes approaching those of transplant recipients [25].

Importantly, our findings offer real‐world insight into the challenges of implementing advanced therapies in middle‐income countries. Treatment decisions were influenced by outpatient feasibility, insurance approval delays, and unequal access to transplant. These realities are rarely reflected in trial settings but shape therapeutic outcomes in much of Latin America. By documenting this experience, we add to the global understanding of how InO may be applied in lower‐resource healthcare systems. We must also note the lack of diagnostic techniques in Latin America for common genes involved in the ABL class, JAK/STAT, or RAS pathways, as well as for genes such as IKAROS and CDKN2.

The adverse event profile was consistent with prior studies. The observed SOS rate of 20% (25% post‐Allo‐HSCT) was slightly higher than historical averages, though most cases were mild and reversible. Only one fatal SOS case occurred. No patient received dual alkylating agents or busulfan conditioning, which are factors associated with higher SOS risk. This supports the idea that careful selection and supportive care mitigated toxicity.

InO's administration in outpatient settings is a major advantage. With ongoing research on subcutaneous blinatumomab [16], future combinations may further reduce hospitalization needs. While blinatumomab may be slightly more cost‐effective overall [26, 27], InO may be better suited to settings where outpatient delivery is essential. New studies explore upfront use of InO in combination regimens [25, 28, 29, 30, 31, 32, 33].

Last, our multivariable analysis emphasized the prognostic value of comorbidity and tumor burden. At the same time, BCR::ABL1‐positive patients showed surprisingly favorable survival, consistent with recent evidence suggesting a better prognosis in the era of TKIs [17, 18, 34]. As noted in the Methods and Conclusion, our modeling strategy sought to assess the stability of these associations across several Cox specifications, while recognizing limitations related to sample size, missing data, and confounding.

This study underscores the feasibility and potential impact of InO in resource‐constrained environments. However, further prospective research is needed to clarify its long‐term efficacy, safety, and integration into public treatment protocols.

## 4 Conclusion

This study presents real‐world experience with InO in patients with relapsed or refractory B‐ALL in Chile, offering insights from a middle‐income healthcare setting where access to advanced therapies remains limited. Our findings align with existing literature, particularly in showing that younger adult patients who achieve complete remission and MRD negativity derive the largest benefit from subsequent Allo‐HSCT. InO proved effective as a bridge‐to‐transplant strategy, especially in contexts where logistical and systemic barriers, such as delays in treatment authorization and variability in transplant availability, complicate care. These results contribute to the global understanding of how novel therapies can be implemented beyond high‐resource environments.

The study's conclusions are limited by a small sample size, short follow‐up, selection bias favoring private‐sector patients, and missing data, all of which constrain generalizability and the strength of inference. Although we applied sensitivity analyzes and multivariable modeling, overfitting and precision loss remain concerns. Nonetheless, these findings support the urgent need for policies that expand access to InO and transplantation, particularly in public health systems. Future studies should prioritize prospective, multicenter designs and cost‐effectiveness analyzes tailored to Latin America to inform clinical and policy decisions around equitable treatment for R/R B‐ALL.

## Author Contributions

**Marcela Espinoza:** conceptualization (equal), formal analysis (equal), investigation (equal), methodology (equal), supervision (equal), writing – original draft (equal), writing – review and editing (equal). **Jorge Rojas‐Vallejos:** data curation (equal), formal analysis (equal), methodology (equal), software (equal), writing – original draft (equal), writing – review and editing (equal). **Nicolás Rodríguez:** conceptualization (equal), investigation (equal). **Gonzalo Guerrero:** conceptualization (equal), investigation (equal). **Miguel López:** conceptualization (equal), investigation (equal). **Natalia Aranguiz:** conceptualization (equal), investigation (equal), writing – review and editing (equal). **Guillermo Conte:** conceptualization (equal), investigation (equal). **Francisco Samaniego:** conceptualization (equal), investigation (equal). **Nicolás Quinteros:** conceptualization (equal), investigation (equal). **Daniel Astete:** conceptualization (equal), investigation (equal). **Lucas Carcamo:** conceptualization (equal), investigation (equal). **Constanza Flores:** conceptualization (equal), investigation (equal). **Ximena Huerta:** conceptualization (equal), investigation (equal). **Mauricio Chandía:** conceptualization (equal), investigation (equal). **Jorge Valenzuela:** conceptualization (equal), investigation (equal). **Marcelo Navarrete:** conceptualization (equal), investigation (equal). **Yorman Flores:** conceptualization (equal), investigation (equal). **Agatha Larrazabal:** conceptualization (equal), investigation (equal), writing – review and editing (equal). **Edgar Zapata:** conceptualization (equal), investigation (equal). **Joaquín Jerez:** conceptualization (equal), formal analysis (equal), investigation (equal), writing – original draft (equal), writing – review and editing (equal).

## Ethics Statement

This study was conducted following the Declaration of Helsinki and approved by the following ethics committees: Scientific and Ethics Committee of the Hospital Barros Luco Trudeau (approval date: December 18, 2024), Scientific and Ethics Committee of Clínica Dávila (April 18, 2024), Scientific and Ethics Committee of Hospital Clínico Universidad de Chile (April 2, 2025), Scientific and Ethics Committee of the Hospital San Borja, part of the Central Metropolitan Health Service (October 4, 2024), Scientific and Ethics Committee of Fundación Arturo López Pérez (January 29, 2025), Scientific and Ethics Committee of the Hospital La Serena (November 12, 2024), Scientific and Ethics Committee of Red Salud Vitacura (June 4, 2024), and Scientific and Ethics Committee of the Hospital Guillermo Grant Benavente (October 10, 2024).

## Conflicts of Interest

The authors declare no conflicts of interest.

## Data Availability

The data that support the findings of this study are available on request from the corresponding author. The data are not publicly available due to privacy restrictions. However, the data may be shared unnamed, not individualized.
