# Correction to “Real‐World Data on Inotuzumab Ozogamicin for Adult Patients With Relapsed/Refractory Acute Lymphoblastic Leukemia: A GRELAL‐Chile Study”

**DOI:** 10.1002/cam4.71385

**Published:** 2025-12-16

M. Espinoza, J. Rojas‐Vallejos, N. Rodríguez, et al., “Real‐World Data on Inotuzumab Ozogamicin for Adult Patients With Relapsed/Refractory Acute Lymphoblastic Leukemia: A GRELAL‐Chile Study,” Cancer Med 14, no. 18 (2025): e71230. doi: https://doi.org/10.1002/cam4.71230.

The error concerns the omission of an additional institutional affiliation for the following authors: Lucas Cárcamo, Agatha Larrazabal, Edgar Zapata, and Joaquín Jerez.

These authors also hold an additional institutional affiliation that was not included in the final published version: Fundación Arturo López Pérez, Santiago, Chile.

The corrected affiliations should read as follows:

Lucas Cárcamo: Hospital Gustavo Fricke, Viña del Mar, Chile; Fundación Arturo López Pérez, Santiago, Chile.

Agatha Larrazabal: Hematology Resident, Universidad de los Andes, Santiago, Chile; Fundación Arturo López Pérez, Santiago, Chile.

Edgar Zapata: Hematology Resident, Universidad de los Andes, Santiago, Chile; Fundación Arturo López Pérez, Santiago, Chile.

Joaquín Jerez: PhD Program in Biomedicine, Faculty of Medicine, Universidad de los Andes, Santiago, Chile; Fundación Arturo López Pérez, Santiago, Chile.

The authors apologize for this error.

